# Risk and protective factors of deliberate self-harm among individuals with substance use disorder: A qualitative study in Pakistan

**DOI:** 10.12669/pjms.40.11.8196

**Published:** 2024-12

**Authors:** Mehak Batool, Sobia Masood

**Affiliations:** 1 Mehak Batool, National Institute of Psychology, Center of Excellence, Quaid-i-Azam University, Islamabad, Pakistan; 2 Sobia Masood, PhD National Institute of Psychology, Center of Excellence, Quaid-i-Azam University, Islamabad, Pakistan

**Keywords:** Self-harm, Substance use, Indigenous predictors, Protective factors, Reflexive thematic analysis

## Abstract

**Objectives:**

This study aimed “To identify the factors which increase the vulnerability of self-harm behavior among individuals with substance use and factors which buffer that risk”.

**Methods:**

The present qualitative study was hosted by National Institute of Psychology, Quaid-i-Azam University, Islamabad, Pakistan. It took seven months, from October 2021 to April 2022 to conduct the study. Face-to-face in-depth interviews were conducted in rehabilitation centers of two different cities. The sample of the study included (*N* = 26) participants, 21 individuals with substance use disorder (with/without deliberate self-harm), and five experts (experienced in dealing with the target population). All the interviews were recorded and transcribed for reflexive thematic analysis. A brief survey was also used to get information about demographics and self-harm behaviors.

**Results:**

The analysis helped to develop nine themes (six risk factors & three protective factors), including separate subthemes for each. The themes were named as a spectrum of adverse experiences, maladaptive personality traits, poor methods of dealing with stressors, chaotic emotions and thoughts about self, internalizing and externalizing problems, and self-harm-related specific factors. Additionally, themes generated for the protective factors were supportive social ties, adaptive coping strategies, and promoting personal resources.

**Conclusion:**

Specific familial, social, and personal factors increase the likelihood of self-harm by causing negative self-evaluation, poor coping skills and psychological distress. This vulnerability ends with self-harm, when combined with conducive environmental situations for the risky behavior. However, the risk can be decreased by providing social and emotional support and encouraging positive mental attitudes.

## INTRODUCTION

Substance use causes considerable economic, societal, and personal costs such as intense levels of self-destructive and health-compromising behaviors. Deliberate self-harm (DSH) and suicide are one of the harmful consequences of substance use. DSH refers to conscious, non-habitual self-harm with/without suicide intent.^1^ Studies show that individuals with substance use are involved in DSH about ten times more often than individuals who do not involve in illicit substance use.^2^ Similarly, the rates of committing suicide are also two to three times greater among this population as compared to men with no substance use disorder.^3^ Many empirical studies have identified the relationship between substance use and DSH; however, evidence is insufficient to understand risk factors beyond prevalence rates.^4^ Therefore, it is crucial to get in-depth understanding and exploration of risk and protective factors for this life-threatening act, especially among the most vulnerable population.

Research suggests that compared with other groups, patients with substance use disorders, irrespective of the type of substance used, are not only at increased risk of self-harm but also repeated and severe forms of DSH have been found among them.^5^ In Pakistan, there are no empirical data related to risk and protective factors of self-harm among substance users, despite the 42% prevalence of substance use among the self-harm-related hospital population.^6^,^7^ Empirical evidence in this area predominantly comes from developed Western countries. Given this context, it was anticipated that the present exploratory study would offer more rich understanding and novel contributions by evaluating the firsthand experiences of the target population. The study targeted only men with substance use disorder as they had higher rates of substance use and suicide in Pakistan (about 78-80%).^8^

The objectives of this study were to understand the experiences and perceptions of individuals with substance use (with/without DSH) and to explore indigenous predictors of DSH among individuals with substance use.

## METHODS

This research employed an exploratory qualitative study design to investigate the objective. The reflexive thematic approach, as described by Braun V et al. and Clarke V et al.^9^ was used with a critical realist epistemological perspective to analyze the data. This approach is based on interpreting participants’ experiences by considering the researchers’ perspectives, contexts, and understanding of the world. The specific approach was useful because the research aimed not to describe the participants’ attitudes or “meaning made” but rather predictors that determine their behavior.^10^

The study was conducted between October 2021 to April 2022. A purposive sampling technique was used to recruit the sample. The sample was accessed from two different drug addiction and rehabilitation centers in Islamabad (*n* = 9; Sunny Trust) and Mandi Bahauddin (*n* = 12; Umeed e Bahaar Rehabilitation Centre). The sample consisted of individuals with substance use disorder (with/without DSH) and experts (experienced in dealing with substance users).

### Ethical Approval:

The present study was approved by ethics committee of National Institute of Psychology, Quaid-i-Azam University, Islamabad on 15^th^ July 2021.

### Inclusion Criteria:

Substance-using participants in the study needed to meet DSM-5 criteria for substance use disorder, they must be of >18 years, and have been in residential treatment for at least three weeks (in order to limit the potential impact of withdrawal symptoms on responses to study questions). Individuals referred by the psychologists who could comprehend properly and having history of self-harm not later than six months (to ensure that respondents could adequately recall the experience) were included in the study. Those without cognitive impairment or psychological disorders (except depressive symptoms) were eligible.

### Exclusion Criteria:

Individuals with any serious physical/mental health (terminal disease/disability) condition were not included.

### Procedure:

Data were collected through in-person interviews lasting between 50 to 115 minutes. A uniform topic guide including open-ended questions based on existing literature was used. Additionally, the study also screened DSH behavior using the first 12 items of the Inventory of Statements About Self-Injury.^11^ The audio-recorded interviews were transcribed following guidelines by Braun and Clarke (2013).^12^ The authors of the study independently coded all the transcripts for purpose of reflexivity. Coding was done by reading transcripts line by line and assigning codes to meaningful statements. Semantic codes were generated when relevant semantic details were interpreted, and latent codes were produced when interpreted useful latent information. The data analysis process was recursive and reiterative; thus, codes were refined over time after the review and discussion of both authors. Themes were generated focusing on “Shared meaning underpinned by a central concept”.^9^ The themes were titled to tell the story about the data. However, where the terminology best fits the meaning, such as “maladaptive personality traits” used that.

The study ensured internal and external validity through an adequate sample size based on the concept of “information power”^13^ and the use of researcher and data triangulation for rich and thick descriptions. During the data analysis, the author’s subjectivity was taken as a resource for interpreting the data rather than simply describing it.^14^

## RESULTS

### Participant demographic characteristics:

A total of 26 individuals participated in the interviews. All the substances using participants (*N*=21) were men with an age range of 20-55 years (*M* = 32.71; *SD* = 10.86). Current substance use was 57.14% heroin, 28.58% cocaine, 4.76% alcohol, 4.76% crystal methamphetamine, and 4.76% cannabis. Whereas the experts’ sample (*n* = 5) included two clinical psychologists, two head of the drug rehabilitation centers and one researcher and clinical psychologist in area of substance use.

**Fig.1:**
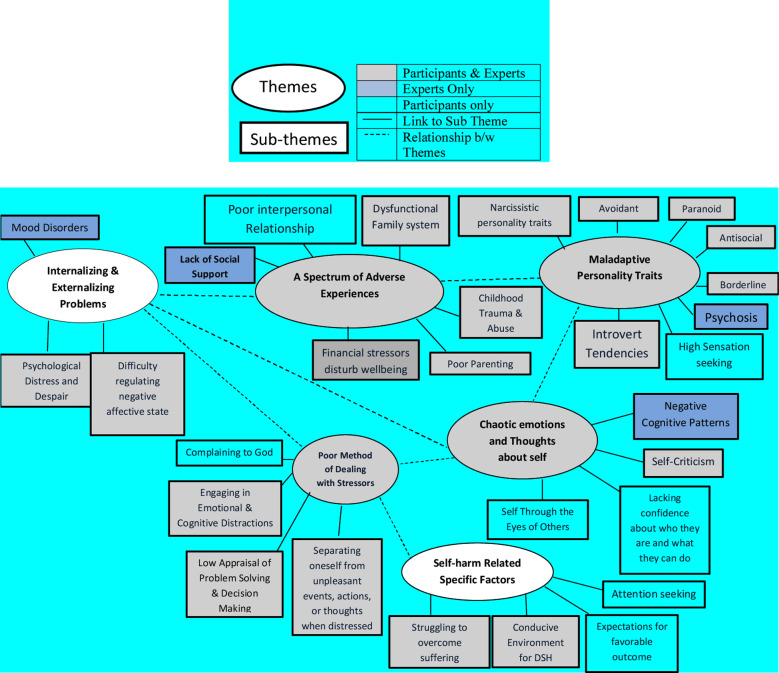
Themes and sub-themes for risk factors of DSH among individuals with substance use.

**Fig.2:**
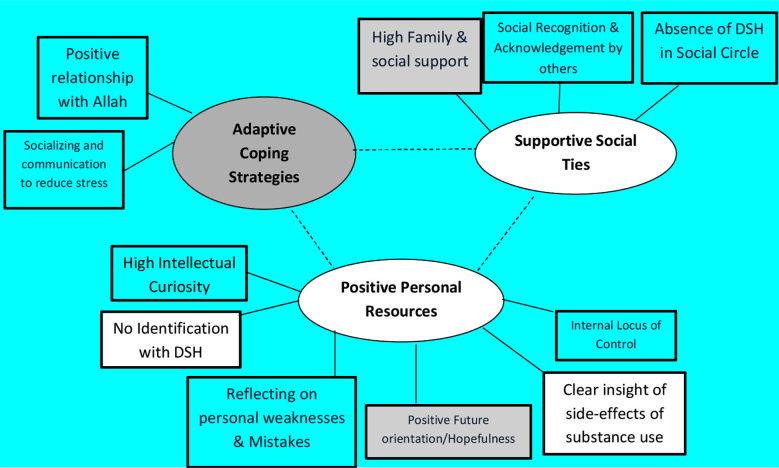
Themes and sub-themes for protective factors of DSH among individuals with substance use.

The reflexive thematic analysis helped to develop nine themes in total, six risk factors (Fig.1) and three protective factors (Fig.2), including separate subthemes for each.

### Theme-1: A Spectrum of Adverse Experiences:

Participants were found to feel very low and sensitive talking about their adversities. Poor communication and lack of affective involvement were reported among the family members of most of the participants. More than half of the participants indicated childhood abuse, as reported by one participant, “In my childhood… well… A couple of the friends I had were not good. When I was new to this field, if even… Even now when… I often face a problem, whenever that thought comes to my mind. Today it has been almost 8, 9, 10 years… almost 10 years have passed. If that comes to mind. So, this causes a little problem. “Did you experience any sexual assault?” yes, yes, I was way younger then. I trusted them but they did bad things (Participant-15).” Another common acute social stressor was a financial difficulty. They were facing problems with finding a job and supporting their family. This was not only a reason for stress but also a reason to neglect of personal health and engage in illicit behaviors.

### Theme-2: Maladaptive Personality Traits:

The dysfunctional family system and childhood adverse experiences give rise to maladaptive personality traits such as a vengeance attitude, impulsiveness, and frequent criminal acts. The theme includes narcissistic, avoidant, borderline, and other maladaptive variants of positive personality traits. Experts also supported this idea by indicating that the second important reason after familial and social factors are personality-related issues that cause a tendency to DSH. The personality traits were interpreted by interpreting participant’s narratives during the interview. Such as, almost half of the participants reported violent acts toward others. One example is: “I have been to jail twice for injuring someone. I stabbed him three times. One hit him in the back. One on his neck and I was about to cut his neck. A boy grabbed me from behind. He said are you crazy… you are going to kill him. I said people die (Participant-9).”

### Theme-3: Inadequate Method of Dealing with Stressors:

The dysfunctional personality characteristics hinder the participants’ ability to deal with life stressors effectively. They lack problem-solving skills and often engage in emotional and cognitive distractions by avoiding problems. As a participant shared, “There is no other solution to my sufferings. I scratch myself. I cut myself and feel relaxed (Participant-2).” They found to be struggling to think clearly and choosing constructive and healthy ways to cope with life stressor. In such states they view self-harm as and ultimate solution to their issues. As stated, “There was no bread or water in the house. There were also financial problems at home. So, what should a person do if he does not harm himself in such tensions (Participant-2).”

### Theme-4: Chaotic emotions and Thoughts about self:

The inability to deal with life problems causes participants to involve in self-criticism. They react harshly and aggressively inwardly, believing that all the problems are due to themselves. Participants are likely to use self-deprecating language, for instance, making negative comments about their potential or apologizing for things that were not their fault. As reported, “Only when I got angry, I used to hit myself with something. I don’t hit anyone rather hit myself. Injure with glass. I tell myself that you don’t need to talk and indulge in an argument when no one acknowledges you? No one listens to you… (Participant-11).” Experts also described that individuals with self-harm usually have negative self-perception and low self-confidence, which hinder their ability to assess their true potentials. Thus, they rely on others’ opinions and feedback for self-evaluation. However, the negative stigmatization by others only exacerbates the problem.

### Theme-5: Internalizing & Externalizing Problems:

They express their feelings either internalizing or externalizing them. They feel further devalued and have low motivation for life. Therefore, they experience depressive symptoms or overly aggressive behavior toward others. As one of the participants stated; “But when I got angry, it is extremely bad……. And when I get angry, I don’t listen to anyone. “So, you never tried to control your anger?” Anger is not controlled. I try but when the anger rises, then it is not controlled. I just want to hurt the next person. I try but I don’t have control (Participant-12).” It shows their lack of ability to regulate negative emotional states and a struggle between anger management and impulse control.

### Theme-6: Self-harm Related Specific Factors:

Having the vulnerability to DSH due to the above-mentioned factors, some specific cognitive and environmental factors like expectations for favorable psychological & social outcomes increase the likelihood of DSH. They found self-harm behavior as the ultimate solution to their problems. “Do you want people to know about your self-harming behavior? Yes… Only then someone will take action in a particular matter. Other will understand the compulsion. When people see blood, then everyone knows what the matter is. Why is he bleeding? That’s why I want everyone to know. When my friends find out, they treat me well (Participant-1)”

### Theme-7: Supportive Social Ties:

The theme generated for the protective factors of self-harm was significant support from family and friends. Individuals without DSH were having friends and loving family members who always stood by them. Such as a participant stated, “My family supports me completely. Whatever happens, they fully support it. My brother also loves me very much. ’Full support… I am proud of my father because he always supported me (Participant-1).” When talking about protective factors of self-harm behavior, experts also pointed out that, “Social support is a very important factor. Family supports provide 50% protection, whereas friends support contributes to 20% safety. Most of the family members do not know how to treat substance-using individuals which ultimately leads to risky behaviors among them (Expert-3).” Supportive relationships hinder feelings of loneliness and provide more tangible assistance in response to daily stressors. Thus, individuals can effectively cope with demanding situations rather than searching for escape through of self-harm.

### Theme-8: Positive Personal Resources:

Participants who proactively seek help for their issues and who have family or social support appeared to have a more positive outlook about themselves and their future thus showing overall positive mental attitudes. They show no identification with self-harm, reflect on personal weaknesses and mistakes, and have an optimistic approach and clear insight into the dark side of substance use. Having positive personal resources, individuals without DSH use adaptive coping strategies to deal with complex and demanding situations.

### Theme-9: Adaptive Coping Strategies:

All the participants stated that expression is a helpful way of managing feelings. Talking to the person causing intense feelings was stated as a valuable coping skill. Additionally, participants reported a more positive religious coping style compared to those with DSH behavior. They always interpret sufferings as familiar to everyone and a hard time that does not stay longer.

## DISCUSSION

This study examines substantial risk and protective factors for DSH among individuals with substance use disorder. It adds to existing literature by providing insights from individuals with substance use disorder and highlights the role of negative family and social factors in DSH behavior. The theme one named as spectrum of adverse experiences includes dysfunctional family system and less gratifying interpersonal relationships. The empirical data similarly outlines that those with DSH have less support, higher levels of rejection, and lower emotional warmth from their parents compared to those without a history of self-harm.^15^ A comparable effect of familial relationships on DSH has been found in Pakistani youth. Such as poor family functioning and interpersonal stress significantly predict self-injury among adults.^16^ The theme also included childhood abuse. Childhood abuse’s psychopathological potential and outcomes are broad. A large proportion of the sample reported physical abuse and few shared experiences of sexual abuse in their childhood. In line with the present findings, the prevalence of DSH is found to be high among those substance users with a history of childhood maltreatment compared to the general population.^17^,^18^ One reason for these findings may be a high level of depression, co-occurring personality disorders,^17^ as well as greater substance use severity^18^ among substance use disorder patients with childhood maltreatment compared to without such experiences. Moreover, as indicated in the subtheme, financial stressors also lead to DSH acts. This theme is unique to the Pakistani population. As Tanweer et al.^19^ found that among Pakistani men, drug use leads to unemployment and poverty. The researcher highlighted that 60% of substance users who were employed before drugs had afterward lost their jobs. Whereas 70% reported facing financial hardship due to drug use. In this way, unemployment and financial hardships, in turn, lead to DSH.

The second theme of dysfunctional personality traits indicates the role of personality is significant in self-harm because DSH occurs due to internally directed motives more relative to interpersonally directed motives. Therefore, DSH is prominent among individuals with borderline and avoidant personalities that share similar underlying motives as DSH i.e., impulsivity and chronic anger.^20^ In Pakistan, personality disorders are one of the significant co-occurring psychiatric conditions with substance use.^21^ Therefore, self-harm acts are prominent among them.

The third theme manifests the importance of coping styles in self-harm. People usually self-injure to cope with elevated psychological distress.^22^ However, not all individuals who experience distress engage in DSH behavior, suggesting underlying psychological processes that may differentiate individuals who self-injure from those who use more positive coping strategies. Pieterse et al.^23^ indicated that among substance-using men, the violent method of DSH is related to an effort to escape from the distressing situation.

According to the fourth theme, negative self-evaluation and low self-esteem predict self-harm behavior. Empirical data confirm these findings by showing that individuals with a recurrent and severe form of DSH have repeatedly been more self-critical than individuals who do not engage in self-harm.^24^ Nagy et al.^25^ also reported that participants in the self-criticism state experience more implicit associations with DSH. Including this, the subtheme of seeing through the eyes of others is also justifiable because stigma towards people with illicit drug use, in turn, leads to the internalization of that stigma by substance users and therefore self-harm.^26^

The fifth theme of internalizing and externalizing problems shows that substance users with self-harm experience high levels of psychological distress, despair, and emotional dysregulation. This is true to Pakistani youth with substance use, as depression is most prominent among them.^27^ Lastly, a theme of self-harm-related specific factors was developed, highlighting the role of increased need for attention to favorable environment for DSH (i.e., self-harm in peer groups and near one’s others), expectations for favorable outcomes, and struggle to overcome adversities. The empirical data shows that if the friends engage in self-harming behavior, this increases the likelihood of DSH acts, mainly when the individual himself experiences internalizing problems and interpersonal distress.^28^ Individuals with DSH behavior in their surroundings usually have positive expectations about self-harming behavior. This identification and DSH-related expectancies increase the chances of prospective self-harm.

In addition to the risk factors, the current study brings out protective factors for self-harm. Findings of a high level of social support protecting against DSH behavior is supported by literature. As Wang et al.^29^ found, a high level of family support and parental cohesion reduce the odd of DSH acts. Likewise, family cohesion and communication in the Pakistani sample have been found to correlate negatively with DSH.^30^ Furthermore, another protective factor for DSH is positive personality traits. This theme agrees with prior research asserting that extroversion, high self-esteem, and conscientiousness play protective roles against self-harm.^31^ Similarly, gratitude and hope, which lower the risk via self-compassion and positive family experience also found high among individuals without DSH.^32^

Another theme showing the buffering effect of DSH is an adaptive coping skill. It is because positive coping styles help better stress management. Therefore, individuals with no history of self-harm use adaptive coping skills, positive religious coping, and focus on the positive aspects of the situation, seeking social support irrespective of the presence or absence of a psychiatric diagnosis.^33^ They show more mindfulness and coping self-efficacy than those with DSH behavior. The mindfulness and more belief in their ability to manage stressful situations in return allows them to select healthier coping alternatives, thus, they are more likely to refrain from self-harm.^34^

The third theme related to protective factors of DSH is named positive personal resources. The subthemes include having no identification with DSH, reflecting on personal weaknesses and mistakes, and having clear insight into the side effects of substance use. Research indicates that people without self-harm have improved mentalizing and mindfulness abilities compared to those with DSH.^35^ Understanding one’s mental state plays a mediating role between childhood maltreatment and DSH.^35^

### Limitations:

The current study provides an in-depth indigenous perspective on risk and protective factors for DSH through data and research triangulation, but causality of predictor variables cannot be determined. The study’s findings might not be generalizable to the community sample as only individuals in residential treatment were included. Moreover, it only included male participants thus, could affect applicability to broader population.

## CONCLUSION

Several predisposing factors make one’s personality vulnerable by increasing negative emotionality and poor self-perception. This hinders one’s potential to effectively deal with life stressors and leads to psychological distress. In this intense psychological and emotional state, when the person observes DSH in the surroundings, DSH is more likely to occur. However, strong social ties, and rational, positive thoughts can buffer the risk of DSH. The study results can be used by clinicians to better understand self-harm and to provide timely interventions to prevent suicide in high-risk populations.

### Authors Contribution:

**MB:** Contributed to conceptualization, methodology, data collection and analysis, as well as manuscript writing and preparation.

**SM:** Helped in data interpretation, critical evaluation and final approval of the manuscript

All authors have read and approved the final version and they are all responsible for the integrity of the study.
